# Determination of the antibiofilm, antiadhesive, and anti-MRSA activities of seven **Salvia** species

**DOI:** 10.4103/0973-1296.71786

**Published:** 2010

**Authors:** Amal G. Al-Bakri, Ghadeer Othman, Fatma U. Afifi

**Affiliations:** *Faculty of Pharmacy, University of Jordan, Queen Rania Al-Abdallah Street, Amman, Jordan*

**Keywords:** Antiadhesion, antibiofilm, MRSA, **Salvia** species

## Abstract

**Background:**

Several **Salvia** species are indigenous to Jordan and are widely used as beverages and spices and for their medicinal properties. The objective of the study was to establish the antimicrobial activities, including the antiadhesive and antibiofilm effects of seven different **Salvia** species.

**Materials and Methods:**

Methods used for planktonic culture included agar diffusion, broth microdilution, and minimal biocidal concentration determination while viable count was used for the determination of the antibiofilm and antiadhesion activities. Overnight cultures of reference strains of **Candida albicans, Escherichia coli, Pseudomonas aeruginosa, and Staphylococcus aureus** and clinical strains of methicillin-resistant **S. aureus** (MRSA) were used as test microorganisms.

**Results:**

An antimicrobial activity toward planktonic cultures demonstrated a significant bacteriocidal activity (≥4 log cycle reduction) for the **S. triloba** extract against **S. aureus** including MRSA. Its volatile oil exhibited an antimicrobial activity covering all tested microorganisms with the exception of **P. aeruginosa**. **S. triloba** extract and volatile oil were successful in preventing and controlling the biofilm, demonstrating antiadhesion and antibiofilm activities, respectively.

**Conclusion:**

These reported activities for **S. triloba** extract and volatile oil allows their listing as potential antibiofilm and anti-MRSA natural agents. This might suggest their use as an antiseptic in the prophylaxis and treatment of **S. aureus**-associated skin infections. The antimicrobial activity of the other tested **Salvia** species was negligible.

## INTRODUCTION

In Jordan, the use of medicinal plants in traditional medicine still plays an important role in health care. Herbal medicine is widely practiced by the nomads and by the herbalists in cities who dispense both locally grown and imported medicinal plants. It is estimated that a large percentage of the Jordanian population still relies on herbal medicine in their daily life.[1–3]

The leaves of **Salvia triloba** are recommended for easing headaches, toothaches, common colds, and digestive problems. It is used as gargle for oral infections and externally for wound healing.[4–6] In hot beverages, **S. triloba** is consumed almost on a daily basis either on its own or as a flavoring agent of black tea.

The antimicrobial activity and their antibacterial potency against planktonic cultures of several **Salvia** species, both medicinal and ornamental, have been demonstrated.[7–13] Nevertheless, previous studies on the antibiofilm and antiadhesion activities of **Salvia** spp. are limited although such studies are of prime importance to demonstrate the capability of an extract to control microbial manifestations associated with living or abiotic surfaces.[14]

World Health Organization (WHO) reports draw attention to the use of herbal medicines as a primary health care source by the vast majority of world’s population, especially in developing countries. This, in turn, reinforces the responsibility of the scientists to devote more attention to the plant kingdom.[15] Moreover, since the high percentage of therapeutically used antimicrobial agents are of natural origin, the interest in investigating plants used in folk medicine with claimed antibacterial activities is a valid quest. With regard to the antimicrobial activity, another important issue is the increasing incidence of antibiotic-resistant strains.[16,17] There is also a growing problem to the phenotypic resistance initiated by their biofilm formation. In the light of these facts, the plant kingdom – and the huge number of constituents in these plants – offers the prospect of a discovery of new potent extracts and bioactive compounds superior to the existing antimicrobial agents.

In the present work, ethanol extracts of seven **Salvia** species, as well as the hydrodistilled volatile oil of **S. triloba**, have been tested for their antibacterial and antifungal activities in planktonic and biofilm cultures.

## MATERIALS AND METHODS

### Plant material

Fresh leaves and aerial parts of the seven different **Salvia** species indigenous to Jordan (*Salvia ceratophylla* L., *S. dominica* L., *S. hierosolymitana* Boiss., *S. indica* L., *S. syriaca* L., *S. fruticosa* Mill. [syn. **S. triloba** L.], and *S. verbenaca* L.) were collected during spring 2008 from Zai, Al-Salt (15 km of north–west Amman). The plant material was identified macroscopically and microscopically using descriptive references.[18] The identification of the plant material was confirmed by Professor B. Abu Irmaileh, Faculty of Agriculture, University of Jordan. Voucher specimens of the plant materials were deposited at the Herbarium of the Department of Pharmaceutical Sciences, Faculty of Pharmacy (reference no. S11-S17). The plants were dried at room temperature and then coarsely powdered.

### Preparation of the plant extracts and volatile oil

Crude plant extracts of the collected seven different **Salvia** species were prepared by refluxing each 2.5-g dried plant in 25-ml 70% ethanol (BDH, UK). The extracts were kept overnight and then evaporated until dryness. Stock concentrations of 10% (w/v; 100 mg/ml) were prepared by dissolving the crude extract in 96% ethanol. The volatile oil of **S. triloba** was prepared by hydrodistillation of the 150-g dry plant material with 2 l water for 2 h using a clevenger apparatus (JSOW, India). Distillation was carried out twice and the oil obtained was pooled, dried over anhydrous sodium sulfate (Na_2_SO_4_), and stored at 4°C in amber glass vials before analysis. The antimicrobial activity of this oil was assessed by adding 100 µl of the oil to a mixture (300 µl) of broth medium and Tween 80 (0.1% v/v). Volatile oil–Tween 80 mixtures were used throughout the study, except for the agar diffusion method where pure oil was used. Thin layer chromatographic (TLC) analysis of the ethanol extract of **S. triloba** was performed according to Wagner and Bladt (1996) using ready coated TLC plates (Merck, Germany).[19]

### Microorganisms and culture conditions

In the present study, overnight cultures of six microorganisms were used. These are **Staphylococcus aureus** ATCC 6538P, **Escherichia coli** ATCC 8739, **Pseudomonas aeruginosa** ATCC 9027, **Candida albicans** ATCC 10231, and two clinical isolates of methicillin-resistant **S. aureus** (MRSA) supplied by Professor A. Shehabi, Faculty of Medicine, University of Jordan. The patient-isolated bacteria were approved by biochemical tests. Bacteria were grown in the nutrient broth medium (Oxoid, UK) while the yeast was grown in Sabouraud medium (HiMedia, India). Batches of the medium (20 ml) were inoculated from fresh culture slopes and incubated overnight at 37°C. Short-term maintenance was done on nutrient agar plates (Oxoid) at 4°C.

### Agar diffusion method

The antimicrobial activity of the plants’ ethanol extracts and **S. triloba** volatile oil was initially assessed against all tested microorganisms using the agar diffusion method as recommended by the Clinical Laboratory Institute (CLSI).[20] Impregnated disks were prepared by the addition of 20 µl plant extract (10% w/v; 100 mg/ml) or **S. triloba** volatile oil (100%) to “susceptibility blank disks” (Oxoid). These were subsequently applied to the inoculated agar plates and then incubated at 37°C for 24 h. Inhibition zone diameters including the diameter of the disk (mm) were measured using a “zone of inhibition” plate reader (Readbiotic; PBI International, Italy). The antimicrobial activity was presented as the inhibition zone (mm) and was calculated using the following equation:

Antimicrobial activity (mm) = Inhibition zone of the tested material - Inhibition zone of Ethanol

### Broth microdilution method (MIC)

MIC of plant extracts and of the **S. triloba** oil against all tested microorganisms was determined using the microdilution method in 96-well plates (Cellstar^®^, Greiner Bio-One, Germany) (NCCLS, 2003). Double-strength medium (100 µl) of the Mueller Hinton broth (Oxoid) (bacterial culture) and Sabauroud medium (yeast culture) were used to fill the first experimental well. The other wells were filled with single-strength medium (100 µl). A volume of 100 µl of the plant extract (10%, w/v) or oil (25%, v/v) was added to the first well. Double-fold serial dilution was then carried out across the plate. The overnight batch culture of the microorganisms (10 µl) was used to inoculate each well to achieve an inoculum size of approximately 1 × 10^6^ CFU/ml. The plates were incubated for 24 h at 37°C. The MIC was calculated according to Al Bakri *et al*.[21] Negative and positive controls were used. Each MIC determination was carried out in triplicate.

Ampicillin, miconazole (both donated by Dar Al Dawa Pharmaceutical Company, Naour, Jordan), and methicillin (Biomedical Inc., USA) were used to assess the accuracy of the MIC method. Chlorhexidine diacetate (Fluka Biochemika, Switzerland) was used as a reference standard biocide, where its MIC values against **S. aureus** reference and clinical strains were determined. The MIC of plant extracts using this method was not feasible due to their turbidity. Subsequently, the antimicrobial activity was assessed using viable count and minimal biocidal concentration (MBC) determination.

### Viable count and minimal biocidal concentration determination

After MIC testing, the 96-microtiter plates set up for the MIC determination were used to determine the viable count and the minimal biocidal concentration (MBC) as described by Al-Bakri *et al*.[21] The MBC value was determined as the lowest concentration showing no detectable growth after incubation (<30 CFU/ml). Positive and negative controls were used. For plant extracts, viable count was performed on the first well devoid of the positive control antimicrobial effect (1.56 mg/ml). Antimicrobial activity at lower concentrations was determined for plant extracts demonstrating a promising activity. For volatile oil, viable count was performed on each well showing no turbidity. The MBC values for chlorhexidine were determined against the reference and clinical strains of **S. aureus**.

### Antibiofilm activity

Antibiofilm activity of the **S. triloba** extract was conducted against the **S. aureus** biofilm, while the antibiofilm activity of the **S. triloba** volatile oil was determined against established biofilms of **S. aureus**, *E. coli*, and *C. albicans*. The antibiofilm activity was determined according to Filoche *et al*. with some modifications.[22] In brief, 100 µl of nutrient medium and 10 µl of microorganism cultures were placed in 96-well plates (Cellstar^®^) and incubated for 24 h at 37°C in order to establish biofilm formation. Following growth (24 h), the supernatant was removed and each well was rinsed with sterile saline for three times. Subsequently, the first experimental well was filled with double-strength nutrient medium while the other wells were filled with single-strength nutrient medium (150 µl). The ethanol extract or volatile oil (150 µl) was added to the first well. Positive and negative controls were used. Double fold serial dilution was then carried out across the plate and plates were incubated for 24 hours at 37°C. After incubation, cultures were removed from wells showing no turbidity and wells were rinsed 3 times with sterile saline to remove unattached bacteria. Attached cells were removed and quantified by viable cell counts. In order to do this, attached cells were removed from wells’ surface using a sterile needle. This step was validated by staining the recovered wells with crystal violet (1%) and visual examination after rinsing 3 times with saline. Viable counts were performed using ten fold serial dilutions. Attached microbial populations were expressed as colony forming unit per well (CFU well^-1^) and the percentage reduction in the viable count (%) was calculated and presented. The antibiofilm activities of **S. triloba** ethanol extract and volatile oil were determined against the two clinical strains of MRSA at concentrations of 0.78 mg/ml and 12.5% v/v, respectively. An antibiofilm activity was contributed to plant extract when the positive control resulted in no antibiofilm activity relative to the negative control. The antibiofilm activities of chlorhexidine towards the established biofilms of tested **S. aureus** strains were evaluated at the corresponding predetermined MBC values.

### Inhibition of biofilm formation (Antiadhesion test)

Antiadhesion activity was determined according to Gursoy *et al*, (2009) with some modifications.[23] Sterile 96 well plates (Cellstar^®^, Greinerbio-one, Germany) were used as the test surface to perform antiadhesion studies. To each well, a volume of 200 µl medium was added. Overnight cultures of the tested microbial strains were used to inoculate each well with 10 µl to give a final inoculum size of approximately 5 × 10^6^ CFU/well. Ethanol extract or volatile oil was added in a predefined subinhibitory concentration to the culture setup and the plate was incubated at 37°C for 24 h. After incubation, the antiadhesion activity of the tested compounds was assessed using the viable counting method. The antiadhesion results were presented as percentage reduction (%) values. Subinhibitory concentrations were determined by performing viable counting. Positive and negative controls were used.

## RESULTS

TLC analysis of the **S. triloba** ethanol extract indicated the presence of flavonoids as the major nonvolatile constituents. GC analysis of the hydrodistilled volatile oil indicated the presence of 1, 8-cineol and camphor as the major constituents.

Initially, the antimicrobial activity of all **Salvia** plant extracts was evaluated using the agar diffusion and viable count methods against all tested microorganisms. Only the **S. triloba** extract exhibited an antimicrobial activity toward **S. aureus**. Consequently, the MBC value for the **S. triloba** plant extract was determined against this microorganism. Table 1 presents the results of the antimicrobial activity of the different **Salvia** plant extracts against **S. aureus**.

**Table 1:** Antimicrobial activity of plant extracts presented as zone of inhibition, viable count, and MBC

Tested agent	Zone of inhibition (mm)	Viable count^a^ (CFU/ml)	MBC (mg/ml)
**	S. aureus	S. aureus	S. aureus
*Ethanol*^b^	7	3.4 × 10^7^	NT^c^
*S. ceratophylla*	0	3.6 × 10^7^	NT^c^
*S. dominica*	1	1.6 × 10^7^	NT^c^
*S. hierosolym*	0	3.8 × 10^7^	NT^c^
*S. indica*	0.5	3.1 × 10^7^	NT^c^
*S. syriaca*	1	3.3 × 10^7^	NT^c^
**S. triloba**	5	ND^d^	1.0 ± 0.35
*S. verbena*	0	3.1 × 10^7^	NT^c^

Results are presented for microorganisms showing susceptibility. Each experiment was performed in triplicate

a Viable count was performed at a plant ethanol extract concentration of 1.56 mg/ml

b Positive contro

c Not tested

d No detectable growth (<30 CFU/ml)

Due to the relatively high antimicrobial activity of the **S. triloba** ethanol extract, the antimicrobial activity of its volatile oil was assessed. Results demonstrated that the **S. triloba** volatile oil exhibited a broad spectrum of activity covering all tested microorganisms, with the exception of **P. aeruginosa** [Table 2]. The efficiency of the **S. triloba** ethanol extract and its volatile oil against MRSA was less comparable to the reference strain where the **S. triloba** ethanol extract resulted in a 4–5 log cycle reduction in the viable count while the volatile oil recorded MIC and MBC values of 2.3% and 9.4%, respectively.

**Table 2:** Antimicrobial activity of the **S. triloba** volatile oil presented as the zone of inhibition MIC, and MBC

Tested microorganism	Zone of inhibition^a^ (mm)	MIC % (v/v)	MBC % (v/v)
**S. aureus**	9.5	0.29 ± 0.00	1.17 ± 0.00
*E. coli*	9.5	0.22 ± 0.07	0.44 ± 0.14
**P. aeruginosa**	0	NA^b^	NA^b^
*C. albicans*	15	0.29 ± 0.00	0.59 ± 0.00

Each experiment was performed in triplicate

a Volatile oil concentration tested was 100%

b No activity

**Figure 1:**
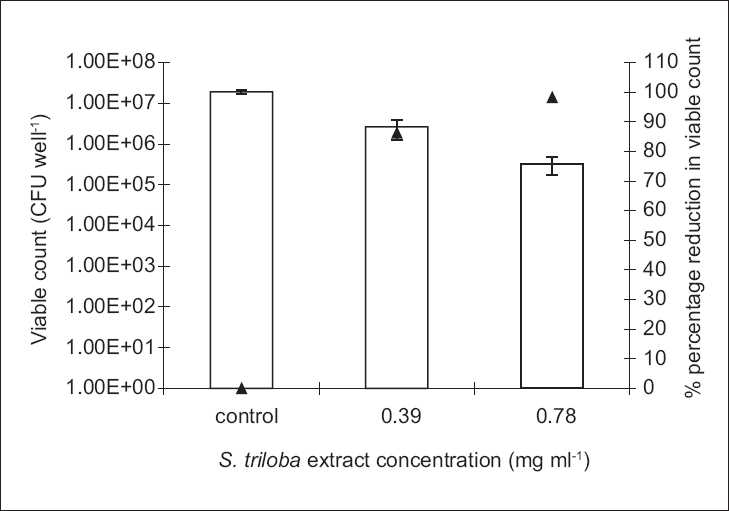
The antibiofilm effect of the ethanol extract of **S. triloba** at different concentrations (mg/ml) presented as CFU/well (empty bars) and percentage reduction (%, ▲) against **S. aureus**. The results are the mean (*n* = 3) ± SD

**Figure 2:**
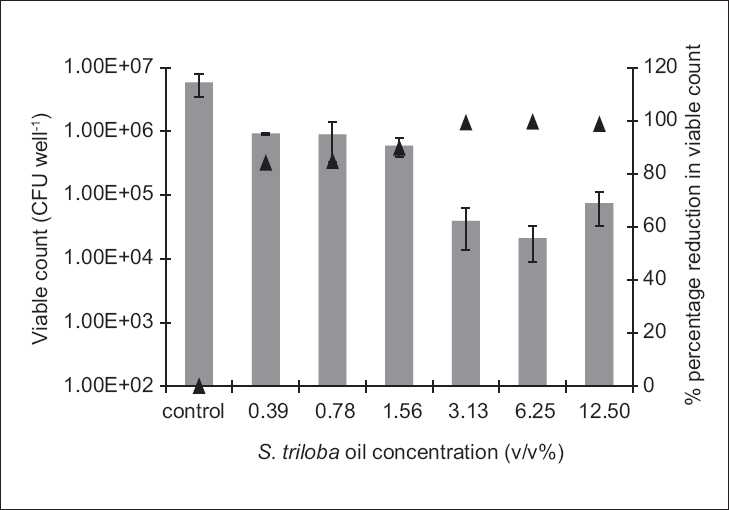
The antibiofilm effect of the volatile oil at different concentrations (v/v %) presented as CFU/well (solid bars) and percentage reduction (%, ▲) against **S. aureus**. The results are the mean (*n* = 3) ± SD

**Figure 3:**
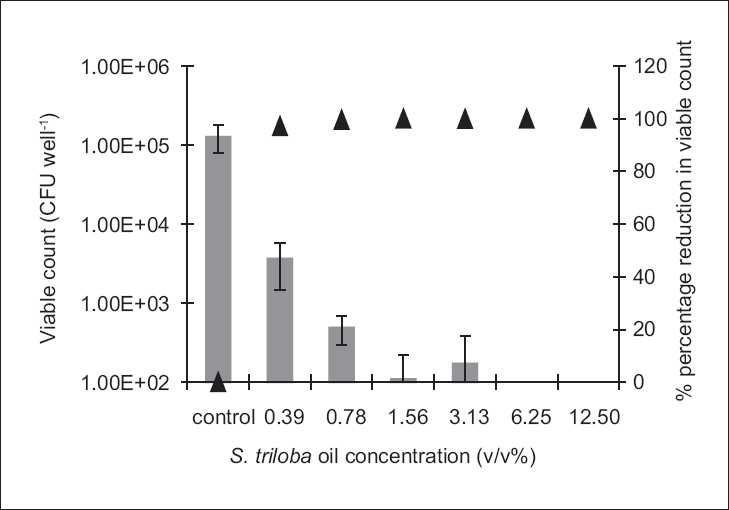
The antibiofilm effect of the volatile oil at different concentrations (v/v %) presented as CFU/well (solid bars) and percentage reduction (%, ▲) against *C. albicans*. The results are the mean (*n* = 3) ± SD

**Figure 4:**
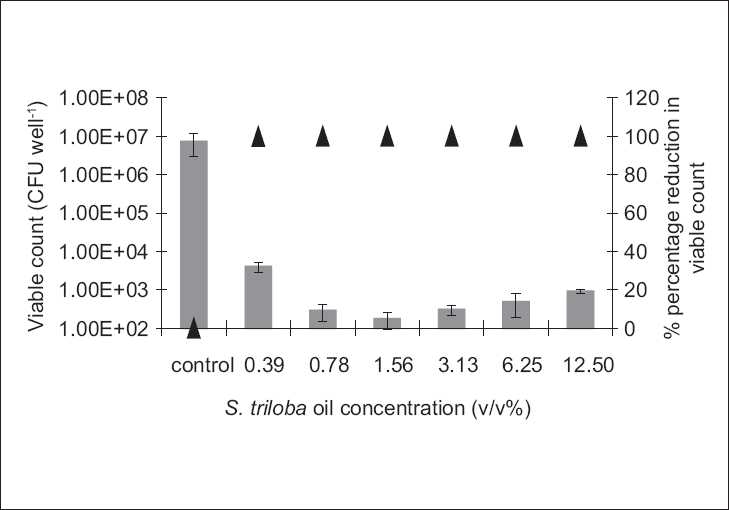
The antibiofilm effect of the volatile oil at different concentrations (v/v %) presented as CFU/well (solid bars) and percentage reduction (%, ▲) against *E. coli*. The results are the mean (*n* = 3) ± SD

The antibiofilm study was only conducted on the microorganisms demonstrating susceptibility to the biocidal activity of the tested agent, i.e., to which MBC value was reported under the experimental conditions. Antibiofilm studies demonstrated a dose-dependent activity [Figures 1–4].

The **S. triloba** ethanol extract resulted in about 1.5 log cycle reduction in **S. aureus** [Figure 1], while its volatile oil exhibited a reduction as high as 4 log cycles against *E. coli* and *C. albicans*, and 2 log cycle reduction against **S. aureus** [Figures 2–4]. Both, plant extract and volatile oil, demonstrated an antibiofilm activity against MRSA clinical strains. The activity of the plant extract against MRSA was lower than that against **S. aureus** whereas **S. triloba** extract, at the same tested concentration (0.78 mg/ml), exhibited an 86.2% and 83.4% reduction against MRSA strains and a 98.3% reduction against **S. aureus**. On the other hand, the antibiofilm activities of the **S. triloba** volatile oil at a concentration of 12.5% obtained against MRSA strains (99.8% and 94.3%) was comparable to that of **S. aureus** (98.7%).

In the present study, chlohexidine was used as a positive control for the MCB and antibiofilm activity determination. The determined MBC value for chlorhexidine against both the reference strain of **S. aureus** (ATCC 6538P) and one of its clinical strains was 62.5 µg/ml versus 31.3 µg/ml, respectively. Results for the antibiofilm activities obtained as a percentage reduction in the population at these chlorhexidine concentrations against the reference and clinical strains of **S. aureus** were 99.6%, 97.4%, and 99.1%, respectively.

The subinhibitory concentrations determined for the **S. triloba** ethanol extract and the volatile oil were 2.5 µg/ml and 0.1%, respectively. These findings were, subsequently, used for the determination of their antiadhesion activities. At these concentrations, both demonstrated antiadhesion activities against *E. coli* (66.7% and 76.0%, respectively). No activity was observed against **P. aeruginosa** and *C. albicans*. Moreover, the **S. triloba** extract demonstrated an antiadhesive effect against **S. aureus** (60.9%) and one of the MRSA strains (37.2%) while the volatile oil did not indicate any antiadhesive activity against either strain.

## DISCUSSION

Efforts toward drug discovery and prudent use of antimicrobial agents are the mainstay for overcoming the worldwide problem of microbial resistance.[24–26] One resort for drug discovery is natural products, crude or isolated from medicinal plants. Many plants and their structurally diverse compounds are known for their antimicrobial activities that have been routinely assessed against planktonic rather than biofilm cultures. A microbial biofilm is ubiquitous in nature and is characterized by its recalcitrance toward antimicrobial treatment.[27] Microbial growth as a biofilm represents a control problem in many clinical, environmental, and industrial settings. The urgent need for antibiofilm agents is clear. Efforts toward the discovery of successful antibiofilm agents included reevaluation of the antimicrobial activity of many known antibiotics, biocides, plant extracts, and natural compounds toward sessile populations.[21] Studies evaluating the antibiofilm activity of tested agents include assessing the activities against established biofilms and the antiadhesive properties at subinhibitory concentrations as a prophylactic measure toward biofilm formation.[12,14,28,29]

In the present study, the antimicrobial activity of ethanol extracts of seven different **Salvia** species against planktonic and biofilm cultures was assessed. Moreover, since the antimicrobial activity of several volatile-oil-containing plants is partially attributed to their volatile oils, the antimicrobial activity of the volatile oils of the most active species was evaluated.[8,10,11,23]

MIC determination using the broth dilution method was not suitable due to plant extracts’ turbidity, making it very difficult to detect any microbial growth. Subsequently, the agar diffusion method, viable count, and MBC were used [Table 1].

Among the tested ethanol extracts, **S. triloba** exhibited the highest antimicrobial activity. Due to differences in the type and concentration of the secondary metabolites across different species of **Salvia** extracts, variations in antimicrobial activities are expected. These quantitative and qualitative differences in constituents are influenced by the chemotypes and, partially, by environmental factors. Method of extraction is another factor that might influence the actual composition.

The antimicrobial activity of the **S. triloba** volatile oil was higher than that of the crude ethanol extract in the extent and spectrum against both, planktonic and biofilm cultures [Tables 1 and 2, Figures 1–4]. This observation correlates with the earlier reports indicating that the potency of several Lamiaceae plants resides in the volatile fraction.[8,10,11,13] In the present study, the actual composition of the **S. triloba** ethanol extract and volatile oil was not determined since several studies demonstrated that crude plant extracts’ bioactivity was superior to that of their purified fractions due to the additive or synergistic activity.[30–32] Also the development of bacterial resistance to multiple plant constituents may be slower than to single entities. This might be considered as another advantage for using extracts rather than pure compounds as antimicrobial agents.[14]

Currently, MRSA is a worldwide problem with limited success with antimicrobial treatment. Accordingly, the reported antimicrobial activity of the **S. triloba** extract, against the sensitive reference strain of **S. aureus**, prompted us to evaluate its activity against MRSA. Our results demonstrated a bacteriocidal activity of the **S. triloba** ethanol extract and its volatile oil against the tested culture of MRSA. The reported results are of significant value and further studies are in progress to evaluate their antibiofilm and antiadhesion activities in combination with nonantibiotic agents.

The prevalent mode of growth in most ecological niches is biofilm. Some plant extracts and purified natural compounds were found to possess antibiofilm and antiadhesion activities.[12,14,28,29] Our findings indicated dose-dependent antibiofilm and antiadhesion activities of the **S. triloba** ethanol extract and its volatile oil. Previous studies on the antibiofilm activities of other **Salvia** species such as *S. pratensis and S. virgata* did not show any effect under the tested experimental conditions.[14] In the present study, the **S. triloba** extract did not achieve biofilm eradication under our experimental conditions. Nevertheless, this was demonstrated by its volatile oil. Moreover, both agents exhibited antiadhesive activities which count as a prophylactic measure toward biofilm establishment.

In comparison to chlorhexidine, the antibiofilm results of the **S. triloba** extract and its volatile oil demonstrated their effectiveness as antibiofilm agents at the tested concentrations where they have achieved considerable antibiofilm activities in comparison to that achieved by chlorhexidine. At predetermined MBC concentrations, chlorhexidine was capable to eradicate the planktonic cultures of **S. aureus**. At these concentrations, the best antibiofilm activity achieved by chlorhexidine was a 2 log cycle reduction. On the other hand, the antibiofilm activities reported in this study reached in some cases to 1–2 log cycle reduction in the population representing a significant effect relative to that of chlorhexidine.

## CONCLUSIONS

In many situations, it is very difficult to eradicate and even to treat microbial biofilms. Hence, the inhibition of microbial adhesion to surfaces and the subsequent biofilm development would be of great value. The present study demonstrated that the **S. triloba** extract and its volatile oil should be considered of great value in preventing and treating biofilms due to their antibiofilm, antiadhesion, and anti-MRSA activities. Furthermore, these findings revealed that plants used in traditional medicine are still one of the significantly reliable sources in discovering cheap, safe, and easy accessible medicines. Based on the demonstrated activity of the **S. triloba** extract against **S. aureus** [Figure 1], it can be suggested that this plant extract might be used as an antiseptic in the prophylaxis and treatment of **S. aureus**-associated skin infections. In conclusion, although **Salvia** species have been previously demonstrated to possess antimicrobial activities against planktonic bacterial cultures, this is the first study to demonstrate the antibiofilm and antiadhesion activities for one of the **Salvia** species, namely, **S. triloba**. Accordingly, these findings justify the consideration of the **S. triloba** extract and oil as potential candidates in successful treatment of skin infections caused by **S. aureus** biofilms.
